# Rational Design of Ligands with Optimized Residence Time

**DOI:** 10.1021/acsptsci.4c00740

**Published:** 2025-01-14

**Authors:** Paolo Carloni, Giulia Rossetti, Christa E. Müller

**Affiliations:** † Computational Biomedicine, Institute for Neuroscience and Medicine, INM-9, Forschungszentrum Jülich GmbH, 52428 Jülich, Germany; ‡ Faculty of Mathematics, Computer Science and Natural Sciences, RWTH Aachen, 52062 Aachen, Germany; § Juelich Supercomputing Center (JSC), Forschungszentrum Jülich GmbH, 52428 Jülich, Germany; ∥ Department of Neurology, Faculty of Medicine, RWTH Aachen, 52074 Aachen, Germany; ⊥ PharmaCenter Bonn, Pharmaceutical Institute, Pharmaceutical & Medicinal Chemistry, University of Bonn, An der Immenburg 4, 53121 Bonn, Germany

**Keywords:** residence time, ligand design, molecular dynamics

## Abstract

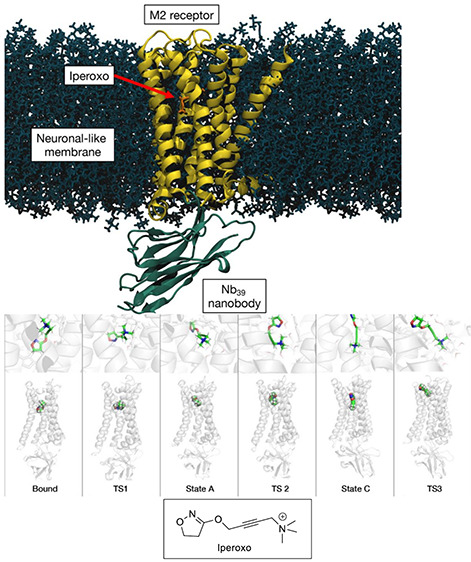

Residence time (RT) refers to the duration that a drug remains bound to its target, affecting its efficacy and pharmacokinetic properties. RTs are key factors in drug design, yet the structure-based design of ligands with desired RTs is still in its infancy. Here, we propose that a combination of cutting-edge molecular dynamics-based methods with classical computer-aided ligand design can help identify ligands that bind not only with high affinity to their target receptors but also with the required residence time to fully exert their beneficial action without causing undesired side effects.

The kinetics of drug unbinding from proteins is a critical factor influencing drug efficacy.^1−3^ Drug–protein target residence time (RT), defined as the reciprocal of a drug’s dissociation rate constant *k*_off_ (RT = 1/*k*_off_), is gaining recognition as a crucial parameter for determining clinical efficacy.^4^ Drugs with relatively long residence times (e.g., of minutes to hours at 37 °C) often show prolonged pharmacodynamic effects^5^ and, in some cases, reduced toxicity, which is beneficial for designing more effective therapeutics.^6^ A long residence time is desired for many drugs, e.g., those targeting chronic diseases such as cancer.^7^ Recently, there has even been an upsurge in the development of infinite residence time covalent drugs for cancer, infection, and other indications.^8^ Long residence times mean that drugs are still active even when they are no longer in circulation.^7^ On the other hand, a drug with a short residence time may in some cases offer advantages such as a reduced risk of prolonged side effects, easier dosage adjustment, and faster reversibility of its effects.^9^

Unfortunately, to elucidate how small changes in drugs’ chemical structures can have profound effects on their RTs has in most cases remained speculative or elusive. It would clearly be of great importance to design drugs that are optimized not only for initial binding affinity but also for prolonged action at the protein target site. This could be achieved by focusing on high free energy intermediates along ligand unbinding pathways, which in turn can be predicted by a vast arsenal of powerful computational tools, and then tested experimentally. Indeed, to rationally design drugs with improved residence times, one must know the structural determinants of the transition state associated with the protein–ligand complex during the unbinding process. This represents the highest free energy configuration along the unbinding pathway, where the ligand is in a transient, less-stable position as it detaches from its target. Ligands that stabilize the intermediate states occurring during dissociation of the ligand from its target are expected to display prolonged RTs. While experimental techniques usually do not reveal the structural details of the transition states, a variety of computer simulation approaches effectively predict the kinetics of drug unbinding at the molecular level and provide a quantitative estimate of the residence time:^10−12^ (i) Very long molecular dynamics (MD) simulations on dedicated machines such as Anton (https://www.deshawresearch.com/) have described this process at the molecular level, and (ii) techniques like infrequent metadynamics, Gaussian Accelerated MD, scaled MD, and dissipation-corrected targeted MD apply biasing potentials to reduce the free energy barriers that slow down dissociation events. These biases artificially accelerate the unbinding process, allowing faster sampling of dissociation events. Correction terms are then used to convert the biased dissociation rates into unbiased residence time estimates. Figure 1 shows an application using one of these methods (infrequent metadynamics) on a neuronal receptor of high medical relevance, the muscarinic receptor M_2_.^13^ (iii) Methods like weighted ensemble and milestoning focus on generating an ensemble of unbiased trajectories by restarting simulations from specific configurations that are more likely to lead to unbinding. This approach increases the probability of observing the dissociation events without directly applying biasing forces, offering a rigorous way to compute unbinding kinetics. (iv) Markov State Models (MSMs), by analyzing molecular simulation data, provide insights into the metastable states of a system and the transition rates between them. MSMs help describe the complex conformational landscape of protein–ligand systems, offering a detailed understanding of both the binding and unbinding processes. Within the limitations associated with the force field used,^13^ one can choose any of these methods to bridge the gap between experimental kinetic data and the detailed molecular mechanisms that underlie drug–target interactions. By stabilizing the transition states of known ligands by chemical modification, one may identify new ligands with longer residence times. Small changes in the interactions that stabilize this transition state can have a significant impact on the rate of dissociation.

**Figure 1:**
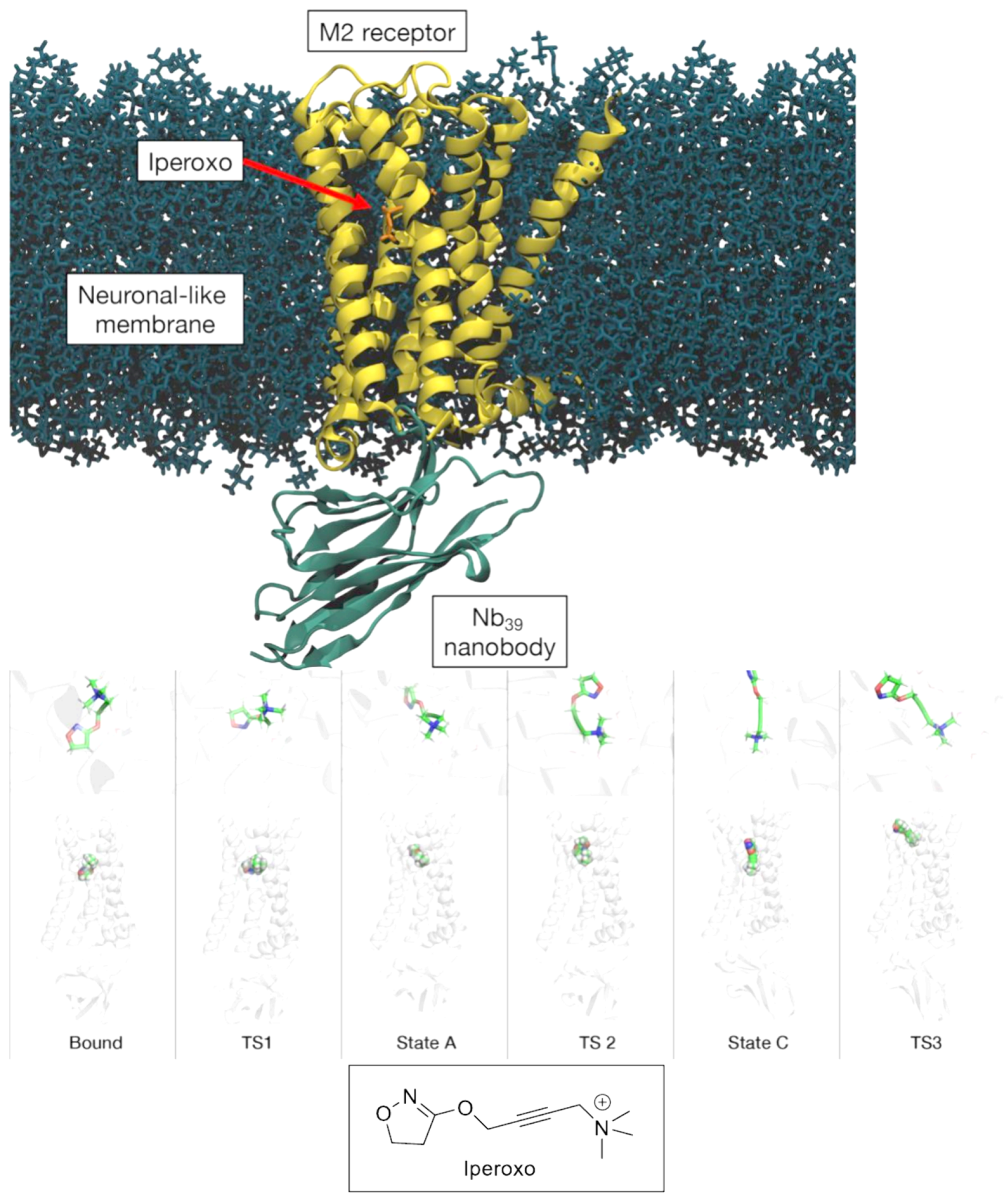
The residence time of a ligand (here the molecule iperoxo) unbinding from its target receptor (the transmembrane G protein-coupled muscarinic receptor M_2_), as investigated by metadynamics simulations.^13^ Top: Simulation setup. Bottom: The simulation explores the bound state and three different intermediate states (State A-C), along with the transition states among them. TS 2 is the state at the highest free energy. Taken from ref (13).

A drug design protocol for ligands with improved residence times could thus involve the following steps: (i) Determining which amino acid residues and noncovalent interactions are most critical for stabilizing the ligand during the transition state (TS 2 in Figure 1). (ii) Based on the knowledge of the transition state structure, designing new ligands or modifying existing ones to enhance their interactions with the protein during the intermediate state. This might involve adding or modifying functional groups on the ligand to better interact with specific residues or to form new bonds that stabilize the transition state. The predictions could be tested experimentally by chemical synthesis of the ligands, followed by kinetic assays. Techniques like X-ray crystallography^14^ and cryo-electron microscopy^15^ might be further used to capture structural snapshots of the transition state analogs.

In conclusion, transition state design is a powerful concept that can lead to the development of drugs with longer-lasting effects, greater target selectivity, and reduced off-target interactions, and thus less side effects and decreased toxicity. Collaborative efforts are required by computational and medicinal chemists involving associated disciplines such as structural biology and pharmacology. If these efforts are made, we can soon expect concrete results in this field by academic and industrial laboratories all over the world.
